# Molecular Characterization of *Leishmania* Species among Patients with Cutaneous Leishmaniasis in Asir Province, Saudi Arabia

**DOI:** 10.3390/pathogens11121472

**Published:** 2022-12-05

**Authors:** Yasser Alraey, Rasha Alhweti, Hatim Almutairi, Abdulrahman Abdullah Al-Qahtani, Mohammed Ibrahim Alshahrani, Mohammed Hussin Asiri, Abdulrhman Mousa Alhammas, Saeed Jubran Alwagdi, Abdulaziz Alshahrani, Abdulaziz Alouffi, Aymen M. Madkhali, Waleed S. Al-Salem, Ahmed A. Al-Qahtani, Ahmed Saif, Sami Ben Hadj Ahmed, Elyes Zhioua

**Affiliations:** 1 Department of Clinical Laboratory Sciences, Central Research Laboratory, College of Applied Medical Sciences, King Khalid University, Abha 61321, Saudi Arabia; 2 Jazan Veterinary Diagnostic Laboratory, Jazan 45142, Saudi Arabia; 3 Public Health Authority, Riyadh 12212, Saudi Arabia; 4 Vector-Borne and Zoonotic Diseases Administration, Saudi Ministry of Health, Abha 61321, Saudi Arabia; 5 King Abdulaziz City for Science and Technology, Riyadh 12354, Saudi Arabia; 6 Department of Medical Laboratory Technology, Faculty of Applied Medical Sciences, Jazan University, Jazan 45142, Saudi Arabia; 7 Department of Infection and Immunity, Research Center, King Faisal Specialist Hospital and Research Center, Riyadh 11211, Saudi Arabia; 8 Department of Clinical Laboratory Sciences, College of Applied Medical Sciences, Najran University, Najran 66624, Saudi Arabia; 9 Unit of Vector Ecology, Pasteur Institute of Tunis, Tunis 1002, Tunisia

**Keywords:** anthroponotic cutaneous leishmaniasis, zoonotic cutaneous leishmaniasis, *Leishmania tropica*, *Leishmania major*, co-circulation, molecular identification

## Abstract

Anthroponotic cutaneous leishmaniais (ACL) and zoonotic cutaneous leishmaniasis (ZCL) caused by *Leishmania tropica* and *Leishmania major*, respectively, are endemic vector-borne diseases in southern Saudi Arabia. In 2021, an outbreak of cutaneous leishmaniasis occurred in the province of Asir. The main objective of our investigation was to analyze the epidemiological features of CL in southern Saudi Arabia. The ministry of health recorded 194 CL patients between January and December 2021 from the Asir province. Our findings showed that the majority of CL patients (87.1%) originated from the governorates of Khamis-Mushait and Abha. Most of the patients were males (62.3%). While CL affected all age groups, those under 13 years old were the most affected (38.1%). For both genders, CL patients were mostly Saudi citizens (90.7%) compared to non-Saudi expatriates. The majority of CL patients (75.2%) suffered from a single lesion, and the majority of lesions (61.3%) were located on the face. The seasonal prevalence of CL showed two peaks, a small one in July–August and a larger one in March. Of a total of 194 Giemsa slides samples, 188 showed positive amplification of *Leishmania* ITS1 gene. Based on PCR-RFLP and PCR-HMR, 183 patients showed positive amplification of *L. tropica* and five patients showed positive amplification of *L. major*. Phylogenetic analysis revealed a clear distinct separation between *L. major* and *L. tropica* sequences. Our results provided strong evidence of the pre-domination of *L. tropica*, the main etiological agent of ACL in Asir province. We reported for the first time the presence of *L. major*, an etiological agent of ZCL in the study areas. The co-circulation of ACL and ZCL highlighted the complexity of the epidemiology of CL in southern Saudi Arabia, and subsequently, further studies to identify competent vectors and reservoir hosts for the establishment of control strategies are needed.

## 1. Introduction

Leishmaniasis is a neglected vector-borne parasitic disease of public health concern caused by an obligate intracellular parasite belonging to the genus *Leishmania*, which is transmitted to humans through the bite of infected female sandflies during blood feeding [1]. Infections caused by *Leishmania* parasites are major global health problems, with high endemicity in developing countries. This neglected tropical disease affects the health of more than 12 million people worldwide, with two million new cases occurring each year [2,3]. Moreover, the increasing number of coinfections with HIV aggravates the burden of this disease [4]. Depending on both the *Leishmania*-infected vector species and the host immunological responses to the etiological agents, leishmaniasis ranges from asymptomatic to self-healing, advanced muco-cutaneous infection, and eventually fatal visceral leishmaniasis, if left untreated [5]. Recently, leishmaniasis has emerged or re-emerged in many geographical areas generating global health and economic concerns that could affect humans [6], domestic animals [7], and wild animals [8]. Environmental changes, poor sanitation, development of agrarian mega-plans leading to the introduction of new reservoir hosts into communities are considered significant risk factors for leishmaniasis [9,10,11,12].

Globally, cutaneous leishmaniasis (CL) is the most common clinical manifestation. Lesions can be single or multiple depending on the number of infected insect bites and *Leishmania* species [13]. Lesions can last for months or even years before healing, leaving permanent scars. Although CL is generally not fatal, the lesions produced may cause substantial disfigurement and severe distress to infected individuals, with lifelong psychological and social consequences [14]. A variety of dermotropic *Leismania* parasite species causes CL. *Leishmania major* and *L. tropica* are the etiological agents of zoonotic cutaneous leishmaniasis (ZCL) and anthroponotic cutaneous leishmaniasis (ACL) in North Africa, the Middle East, and Central Asia [15,16,17]. In East Africa, CL is mainly caused by *L. aethiopica* [18]. In Central and South America, CL is caused by several *Leishmania* species, including *L. mexicana*, *L. amazonensis*, and *L. venezuelensis* [19]. Cutaneous leishmaniasis caused by viscerotropic parasites, such as *L. infantum* [13] and *L. donovani,* is less prevalent [20]. Cutaneous leishmaniasis is the most common form of the disease in Saudi Arabia. The disease is most prevalent in Al-Hassa oasis, with an outbreak in 1983 reporting 18,000 cases [21]. Since the establishment of the national control program, the incidence has declined [22]. According to the Ministry of Health of Saudi Arabia, the average annual incidence was 2500 cases per year, with Al-Hassa, Al-Madinah, Ha’il, and Al-Qaseem being the most endemic areas [23]. Cutaneous leishmaniasis is spreading to new areas leading to the emergence of new foci [24]. The geographical expansion of CL in Saudi Arabia is mainly related to anthroponotic disturbance of natural ecosystems due to massive urbanization and development of agricultural projects leading to the establishment of a stable cycle of leishmaniasis, including sandfly vectors, rodent reservoirs, and non-immune populations and subsequently increasing risk of transmission [25]. 

It is well known that Asir Province is a major CL focus, and children are at the highest risk of CL infection [24]. A previous study reported that *L. tropica* is the main parasite circulating in Asir province [26]. However, in the aforementioned study, only a small sample size was included in addition to the use of Giemsa stain as a diagnostic tool limited the outcome of this eco-epidemiological investigation. For a better understanding of the epidemiological situation in Asir province, investigating a large number of CL patients and using molecular approaches to identify circulating *Leishmania* species is highly needed. 

## 2. Materials and Methods

### 2.1. Study Area and Sample Collection

**Figure 1:**
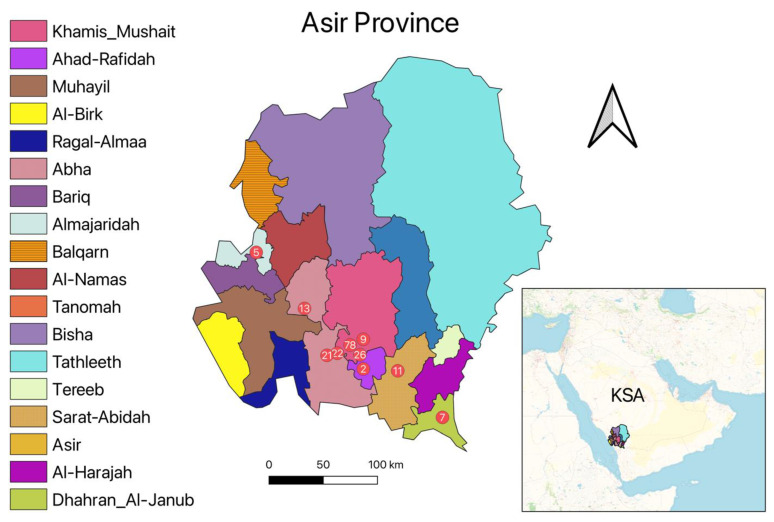
Map of Asir province with the number of CL patients according to their geographical location.

This study was conducted in Asir province located in the southwest of Saudi Arabia (Figure 1). From January to December 2021, Giemsa stain slides were taken from 194 suspected CL patients’ skin lesions. The diagnostic was based on the identification of *Leishmania* parasites within Giemsa-stained slides. Following parasite identification, DNA was extracted from Giemsa-stained slides to identify *Leishmania* species. Demographic information about patients, such as age, gender, residence, lesion location, and number of lesions was also recorded. 

### 2.2. DNA Extraction 

The genomic DNA was extracted from collected Giemsa stain slides using the DNAeasy Blood and Tissue Kit (QIAGEN, Hannover, Germany) according to the manufacturer’s instructions. The extracted DNA was kept at −20 °C for further molecular assays. 

### 2.3. Leishmania Identification Using PCR-RFLP Assay

Two primers were used for DNA amplification of the *Leishmania* internal transcribed spacer 1 target gene (ITS1) of approximately 320 bp LITSR (5′-CTGGATCATTTTCCGATG-3′) and L5.8S (5′-TGATACCACTTATCGCACTT-3′) [27,28]. The 20 μL reaction mixture contained 1× Dream Taq buffer with 2 mM MgCl2 (Thermo Scientific, Walthman, MA, USA), 0.25 mM dNTPs mix, 500 nM of each primer, and 0.125 U of Dream Taq DNA polymerase (Thermo Scientific, USA). The PCR reactions were performed in a T100 Thermal Cycler (Bio-Rad, Watford, UK). Cycling conditions started with an initial denaturation at 98 °C for 2 min, followed by 35 cycles of denaturation at 95 °C for 20 s, annealing at 53 °C for 30, s, and extension at 72 °C for 30 s. This was followed by a final extension at 72 °C for 5 min. After that, the PCR products were digested using the *HaeIII* enzyme. Finally, PCR products were analyzed by gel electrophoresis and stained with SYBR Safe (Invitrogen, Waltham, MA, USA) to improve the DNA visibility under UV light. A positive control with known DNA *Leishmania* species was used to assess PCR efficiency and negative water control to check for any contamination. ITS1-PCR products of the positive samples were purified using the QIAquick PCR purification kit (Qiagen, Valencia, CA, USA) and submitted for sequencing under the forward primer to confirm *Leishmania* species. 

### 2.4. Leishmania Identification Using Real-Time PCR-HRM Assay

The high-resolution melt PCR (PCR-HRM) was used to improve the resolution of *Leishmania* species identification. Two primers (F: 5′-CACGTTATGTGAGCCGTTATCC-3′ and R: 5′-GCCTTTCCCACATACACAGC-3′) were used to differentiate between *L. major* and *L. tropica* [28]. PCR reactions were carried out in an HRM capable of performing out CFX Connect Real-Time PCR Detection System (Biorad, UK). The final volume of 20 μL contained 1× Luna Universal qPCR SYBR Green-based master mix (NEB, Cambridge, UK), 500 nM of each primer. Cycling conditions started with 1 min of denaturation at 95 °C, followed by denaturation at 95 °C for 15 s, followed by annealing and extension at 60 °C for 30 s. After 35 cycles, the HRM was carried out by denaturing at 95 °C for 1 min, then reannealing at 50 °C for 30 s, and gradually raising the temperature by 0.1 °C increments every 2 s while recording changes in fluorescence. Samples with a cycle threshold level (Ct) below 33 were treated as positive amplification of *Leishmania.* Samples with melting temperature values of 84. 2 °C and 85.8 °C corresponded to *L. tropica* and *L. major*, respectively.

### 2.5. Data Analysis

The recorded data were translated into English and digitized in Excel for statistical analysis using R statistical Language v. 4.0.5.

The Open-source software QGIS (Quantum GIS version 3.20.0) was used to map the spatial distribution of CL cases in Asir province in 2021. A Mann–Kendall trend test was used to determine whether or not a trend exists in time series data (monthly CL cases).

### 2.6. Phylogenetic Analysis

The sequences resulted from our samples were added to other similar sequences obtained from a previous *Leishmania* study performed in Eastern Saudi Arabia [29]. Moreover, more sequences were obtained from GenBank by using blast analysis [30]. Details about the analyzed sequences are shown in Table 1. After the alignment of all sequences by using the MAFFT aligner [31], the maximum likelihood fits of 24 different nucleotide substitution models were done to the alignment, and the Jukes–Cantor model was chosen to be fed as a prior when building the tree since it had the lowest Bayesian information criterion score (BIC) and the maximum likelihood value (lnL). The substitution model estimation was done using the MEGAX software [32].

A Bayesian tree was constructed using version v1.10.4 of the BEAST suite [33] with the following prior assumptions: (1) The population size remained constant throughout the time covered by the genealogy, generate a random starting tree under the coalescent process, (2) the Jukes–Cantor substitution model [34], and (3) constant coalescent likelihood with strict clocks (uniform rates across branches) were used as prior and then ran for 10 million iterations. After that, a consensus tree was generated after discarding the first 10% as burn-in using Tree Annotator, which is part of the BEAST suite. The final tree was then visualized and examined using Figtree software [35].

### 2.7. Ethical Approval 

The study was carried out under ethical approval from the Regional Committee for Research Ethics of the ministry of health (Approval Number H-06-B-091).

## 3. Results 

### 3.1. Socio-Epidemiological Features

Of a total of 194 CL patients (121 males and 73 females) from Asir province reported in 2021, 58.2% (N = 113) originated from the governorate of Khamis–Mushait and 28.8% (N = 56) from the governorate of Abha (Table 2). According to reported data, most of the notified CL cases (38.1%, N = 74) were under 13 years of age (34% males and 46% females). For both genders, Saudi citizens were more likely to suffer from CL than non-Saudi expatriates. The majority of CL patients suffered from a single lesion for both males (80%) and females (67%). The majority of lesions were located on the face (58% for females and 64% for males) and hands (29% for females and 25% for males) (Table 2).

### 3.2. Clinical Characteristics of Cutaneous Leishmaniasis

**Figure 2:**
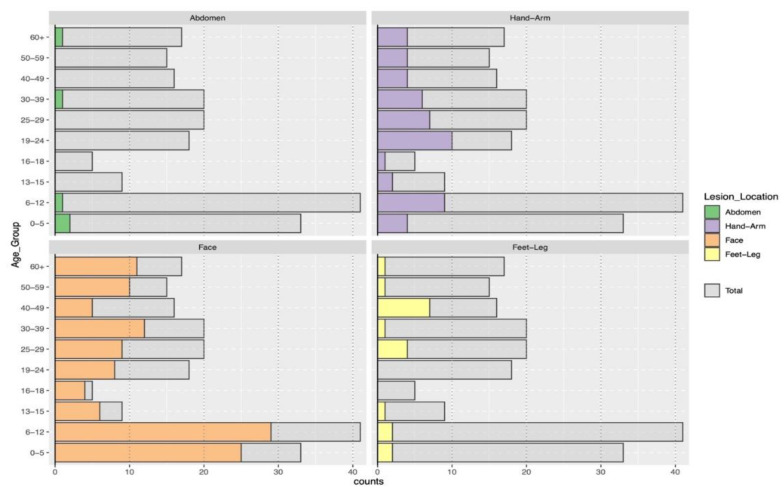
Distribution of lesions in relation to body sites according to age groups.

According to the site of lesions, facial lesions were more common among patients under 13 years old, while lesions on the upper and lower limbs were frequently observed in patients aged 19 to 60 years old (Figure 2). 

**Figure 3:**
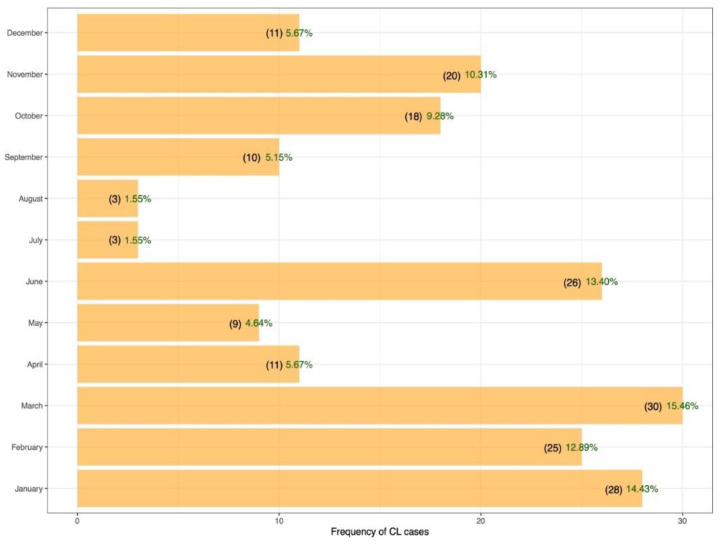
Monthly distribution of cutaneous leishmaniasis cases reported in Asir province during 2021.

The monthly prevalence of CL is variable, with the highest observed in March (15.4%) and the lowest in July–August (1.5%) (Figure 3). However, no significant trend in the monthly variation of CL cases was observed (*p*-value = 0.2415)

### 3.3. Molecular Characterization of Cutaneous Leishmaniasis in Asir Region

**Figure 4:**
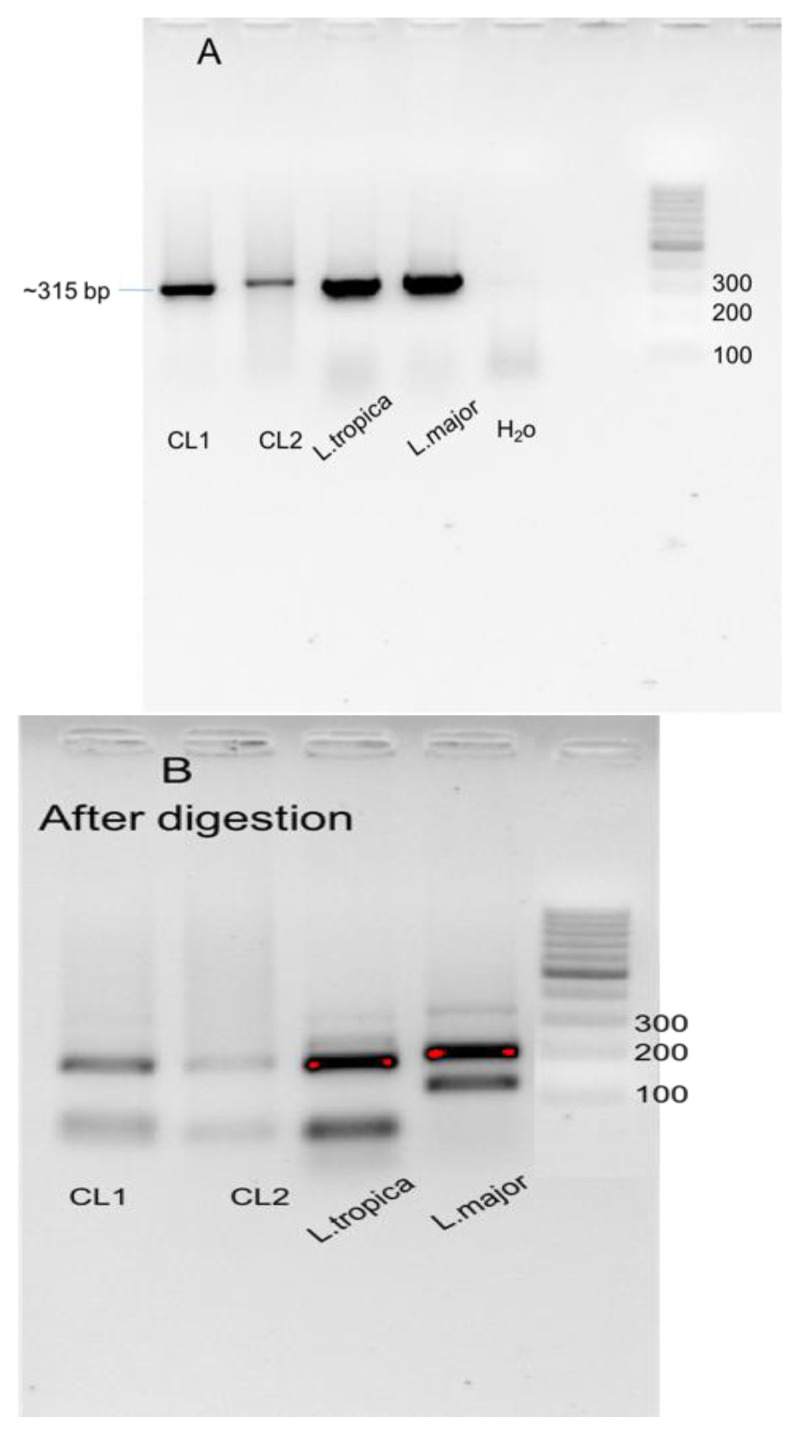
SYBR safe–stained agarose gel showing ITS1 identification. Bands were separated on a 2% agarose gel for 30 min to document differences in RFLP patterns: (**A**) Positive amplification of ITS1 in CL smears 1 and 2. Positive controls have shown positive bands, (**B**) digestion of amplified ITS1 regions of *Leishmania* species with *HaeIII* enzyme. CL1 and CL2 showed positive results corresponding to *L. tropica*.

**Figure 5:**
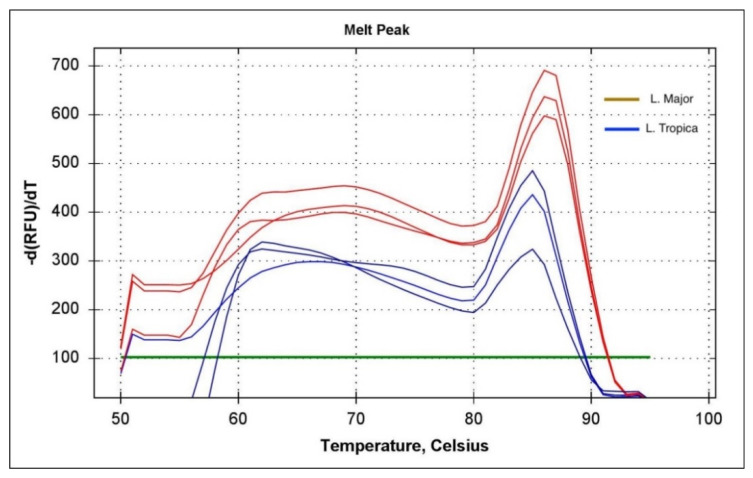
Real-time PCR-HRM analysis of *Leishmania* infection in cutaneous leishmaniasis human samples. The green line is the threshold line that was set to ignore noising peaks below 100.

Out of 194 Giemsa slides samples, 179 showed positive amplification of *Leishmania* ITS1 gene (Figure 4). Based on PCR-HMR, 183 patients showed positive amplification of *L. tropica,* and five patients showed positive amplification of *L. major*, yielding a total of 188 positive samples (Figure 5). It is important to note that of a total of 188 positive samples using conventional PCR, 26 showed very weak amplification and, therefore, very weak bands on the gel after RFLP step. As a result, it was difficult to determine the species of *Leishmania* presented in the sample, which then was confirmed by PCR-HMR. Moreover, 9 of 188 samples showed negative results with PCR-RFLP (no bands). These nine samples tested positive by PCR-HMR (cycle Ct = around 29 cycles).

### 3.4. Phylogenetic Analysis

**Figure 6:**
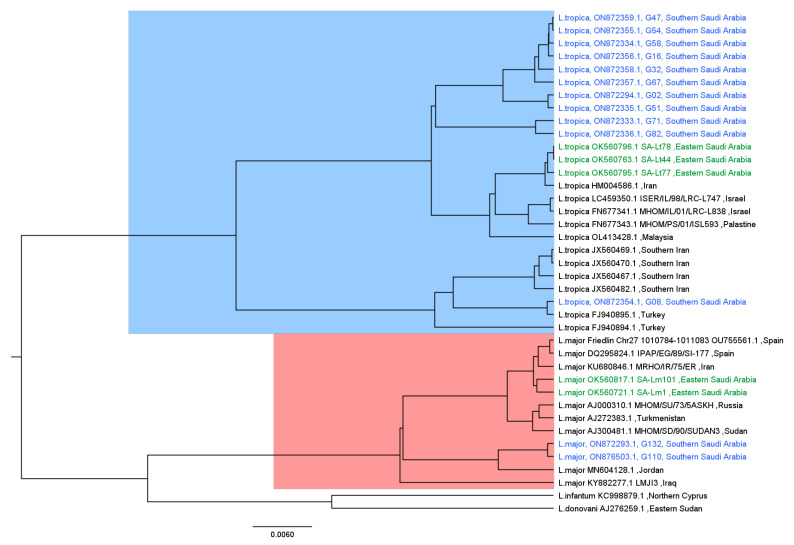
Bayesian phylogenetic tree generated with 39 ITS1 sequences. Our sequences are colored in blue, whereas sequences from eastern Saudi Arabia are colored in green, and the remained sequences from other countries are colored in black.

For the phylogenetic analysis, we selected samples with strong bands based on geographical location. Out of a total of 188 positive samples, only 13 sequences were analyzed. Phylogenetic analysis revealed a clear distinct separation between *L. major* and *L. tropica* sequences (Figure 6). All sequences of *L. tropica* were clustered together except for one sample (G08), which was taken from a non-Saudi patient, a 36-year-old Sudanese man, and appeared to be grouped with sequences from outside of Saudi Arabia. The Eastern Saudi Arabia sequences were also more clustered with sequences from outside Saudi Arabia. Similarly, *L. major* sequences were relatively clustered together and more grouped with sequences from Jordan. However, Eastern Saudi Arabia sequences were more clustered with more diverse sequences. Moreover, the outgroup sequences from *L. major* were *L. infantum* and *L. donovani*.

## 4. Discussion

A total of 1565 CL cases were reported from the Asir province during 2011–2020 [24], and subsequently, this province is considered endemic for CL. It is of major epidemiological importance to point out that due to under-reporting, the actual number of CL patients in this region is much higher, and subsequently, the percentage of patients evaluated in this study is low. While several cases are annually reported from the Asir province, the epidemiology of CL has not been well documented. A relatively recent study showed the predominance of *L. tropica* in Asir region [26]. However, the aforementioned study is focused on different regions of Saudi Arabia, with a limited sample size in each one. The objective of the present work was to identify circulating *Leishmania* species and to perform a comprehensive study on the epidemiological features of CL in Asir region.

The majority of patients were male (62.37%, N = 194) compared to female (37.62%). The high prevalence of CL among males could be attributed to nightly outdoor activities compared to females. Furthermore, females conventionally cover the outermost portions of their bodies, thus protecting them from infected bites by sandflies. The findings of previous studies performed in Saudi Arabia and in other countries from the Middle East, North Africa, and Central Asia showed a predominance of CL cases among males [36,37,38,39,40], and subsequently, they are in agreement with our results. Only one study reported similar CL prevalence between males and females in Saudi Arabia [21].

The geographical distribution of CL clusters in space with the highest prevalence was observed in the governorates and Abha and Khamis-Mushait. The predominance of cutaneous leishmaniasis caused by *L. tropica* in the south west of Saudi Arabia is related to the distribution of *Ph. sergenti*, the main vector of *L. tropica* [41]. However, infection with *L. major* is more prevalent mainly in the northwest, the center, and the east of Saudi Arabia, where *Ph. papatasi* is the main vector [26,37]. Similar findings were reported in Tunisia, where infections with *L. major* and *L. tropica* are prevalent in the center and in the southwest, respectively [15,42].

While *L. tropica* infection is mostly located on the face with a single lesion, infection with *L. major* is located on the limbs with multiple lesions [13]. In the present study, we showed a predominance of *L. tropica* compared to *L. major*, and therefore, common cases presented with a single lesion, with the face being the most commonly affected site, followed by the hands. Previous studies performed in Saudi Arabia and in North Africa reported similar trends with the majority of cases presenting with a distinct lesion on the face [15,21]. Conversely, the study conducted in Al-Madinah district indicated that the majority of patients have more than one lesion and the majority of them were observed in the lower extremities. Lesions’ location and their number on the body sites are dependent on the *Leishmania* parasite species and its vector [13].

The highest prevalence of CL was observed among children under 13 years old. This finding could be explained by the fact that this age group has no previous exposition to sandfly bite, and subsequently, it is the most naïve population in the community. Another study performed in Al-Madinah reported that CL is prevalent in all age groups [38]. However, Amin et al. [22] reported that CL cases from Central Saudi Arabia were mainly reported in the age group of 15–45 years. Similar results were reported in the northwest of Saudi Arabia and in Central Tunisia [36,43].

Taking into account that the number of expatriates involved in the present study is low compared to Saudi Citizens, it is expected that the majority of the cases were among Saudi residents. Similar results were reported by Al-Tawfiq and AbuKhamsin [21], showing a predominance of CL among Saudi citizens (98.3%). Another study reported similar CL prevalence between Saudi citizens and expatriates. [22,36,37]. However, previous studies performed in Arar have shown that CL prevalence was higher among expatriates compared to Saudi citizens [44]. Hence, CL prevalence is not related to nationalities but rather to the exposition site, the level of awareness, and subsequently, the protection against the vector. In addition, CL prevalence is limited by the under-reporting of the disease among communities.

The monthly variation of CL prevalence is the highest in January through March and the lowest in July–August. Cases of CL patients in the northeastern Saudi Arabia were reported in all months of the year with a minimum in June–July [36]. In CL endemic areas located in the East of Saudi Arabia, the number of cases showed a steep increase starting from November, reached a peak during January and February, and then declined by March and April [22]. In Tunisia, lesions that emerged during June–January were caused mostly by *L. major* (64.7%), and lesions that emerged during February–May were caused mainly by *L. tropica* [15].

An entomological investigation performed in the Al Baha region located between Makkah and Asir regions in southwestern Saudi Arabia showed that *Phlebotomus bergeroti* is the most abundant sandfly species, followed by *Ph. sergenti*, and *Ph. papatasi* [45]. The infection prevalence of field-collected *Ph. papatasi* and *Ph. sergenti* from the northwestern Saudi Arabia with *L. major* and *L. tropica* were 23.7% and 31%, respectively [46]. The fat sand rats *Psammomys obesus* and the hyrax, reservoirs of *L. major* and *L. tropica*, respectively [47,48], are present in Saudi Arabia [49]. The aforementioned studies provide strong evidence that the co-circulation of *L. tropica* and *L. major* in CL patients from the Asir province is caused by a zoonotic transmission of both *Leishmania* species involving sandfly vectors (*Ph. tropica* and *Ph. papatasi*) and rodent reservoirs (hyrax and fat sand rats). Hence, investigations on sandfly vectors and potential rodent reservoirs of *Leishmania* species in Asir province are highly needed.

The monthly variation of CL prevalence is related to the seasonal sandfly activity, the seasonal sandfly infection with *Leishmania* parasite, and the incubation period of the disease [50]. *Phlebotomus sergenti* is present in all months of the year, with one major peak in May–June [51]. The seasonal activity of *Ph. papatasi* is bimodal, with a large peak in May–June and a small one in August [45]. Further studies are needed to investigate the monthly variation of CL prevalence caused by *L. tropica* and *L. major* in Asir province.

The predominance of cutaneous leishmaniasis caused by *L. tropica* in southwestern Saudi Arabia is related to the distribution of *Ph. sergenti*, the main vector of *L. tropica* [41]. However, infection with *L. major* is more prevalent mainly in the northwest and in the center of Saudi Arabia, where *Ph. papatasi* is the main vector [37]. Mixed foci were reported in northwestern and central Saudi Arabia [26,52].

While a larger sample size might reflect the phylogenetic relatedness better than a small collection, sequencing all positive samples from 17 governorates in Asir province was not feasible due to financial constraints. The phylogenetic analysis of the selected positive samples showed that *L. tropica* and *L. major* clustered in separate clades, distinct from the *L. donovani* complex (*L. infantum* and *L. donovani*). Our results provided strong evidence that *L. tropica* is the predominant *Leishmania* species circulating in the investigated areas.

Taking into account that *L. tropica* was the only *Leishmania* species so far isolated from field-collected *Ph. sergenti* in Abha, located in Asir province [41], the dominance of *L. tropica*-infection in CL patients was expected. The anthroponotic form of CL (ACL) caused by *L. tropica* and transmitted mainly by *Ph. sergenti* is endemic in southwestern Saudi Arabia [41]. However, the presence of hyrax in southern Saudi Arabia [50] also suggests a zoonotic transmission of *L. tropica*. Thus, more studies to assess the transmission of *L. tropica* among sandfly vectors, potential rodent reservoir hosts, and humans in Saudi Arabia are highly needed. The occurrence of a few cases of CL caused by *L. major*, an etiological agent of ZCL in small micro-foci might be related to the low abundance of *P. papatasi* in southwestern Saudi Arabia [51]. We report for the first time a mixed focus in southwestern Saudi Arabia. Similar findings were reported from the southwest and the center of Tunisia [15,53]. The epidemiology of CL in southwestern Saudi Arabia is highly complex by the high diversity of sandfly vectors and their associated *Leishmania* species, leading to mixed forms of CL caused by different pathogens. Therefore, a better understanding of the ecology of sandfly vectors is highly needed for efficient control to reduce the indoor abundance of sandfly vectors and subsequently reduce the incidence of CL.

Although Giemsa staining is a primary diagnostic tool, the lack of confirmation of amastigotes in the lesion’s indirect smears and tissue specimens can easily lead to misdiagnosis [54]. PCR analysis of *Leishmania* species is an accurate and effective tool that has been used in leishmaniasis research. This approach has also been used for the taxonomic differentiation of *Leishmania* species because of its high sensitivity and specificity [55]. However, real-time PCR-based amplification of the *Leishmania* ITS1, followed by HRM, was found to be more sensitive in identifying *Leishmania* infections in CL lesions over the ITS1-PCR [28]. Furthermore, this technique was shown to be highly specific in discriminating between *L. major* and *L. tropica* infections based on their corresponding melting temperatures. Thus, combining PCR-RFLP and PCR-HMR for the epidemiological studies of *Leishmania* in CL focus is useful for accurate detection and characterization of the infecting parasites compared to microscopic examination.

## 5. Conclusions

Our results provided strong evidence of the pre-domination of *L. tropica*, the main etiological agent of CL in Asir province, Saudi Arabia. It displays a wide clinical polymorphism and, subsequently, should be considered in strategic planning and future diagnosis, treatment, and control programs. We reported for the first time the presence of *L. major*, an etiological agent of ZCL in the study areas. Moreover, this study highlights a valuable tool of the PCR-HRM assay in selecting optimal therapy and treatment regimens, especially in complex localities where more than one *Leishmania* species is present. Further studies to identify competent vectors and reservoir hosts are needed to clarify the epidemiological situation of CL in Asir province.

## Figures and Tables

**Table 1:** List of all sequences used for phylogenetic analysis with available WHO codes or strain names. Our sequences colored in blue and those of Al-Rashed et al. [29] are colored in green.

Species	Strain Name/WHO Code	Country	GenBank Accession
*L. donovani*		Eastern Sudan	AJ276259.1
*L. infantum*		Northern Cyprus	KC998879.1
*L. major*	MHOM/SU/73/5ASKH	Russia	AJ000310.1
*L. major*		Turkmenistan	AJ272383.1
*L. major*	MHOM/SD/90/SUDAN3	Sudan	AJ300481.1
*L. major*	IPAP/EG/89/SI-177	Spain	DQ295824.1
*L. major*	Friedlin Chr27:1010784-1011083	Spain	OU755561.1
*L. major*	MRHO/IR/75/ER	Iran	KU680846.1
*L. major*	LMJI3	Iraq	KY882277.1
*L. major*		Jordan	MN604128.1
*L. major*	**SA-Lm1**	**Eastern Saudi Arabia**	OK560721.1
*L. major*	**SA-Lm101**	**Eastern Saudi Arabia**	OK560817.1
*L. major*	MHOM/SA/2021/G132	Southern Saudi Arabia	ON872293.1
*L. major*	MHOM/SA/2021/G110	Southern Saudi Arabia	ON876503.1
*L. tropica*		Turkey	FJ940894.1
*L. tropica*		Turkey	FJ940895.1
*L. tropica*	MHOM/IL/01/LRC-L838	Israel	FN677341.1
*L. tropica*	MHOM/PS/01/ISL593	Palestine	FN677343.1
*L. tropica*		Iran	HM004586.1
*L. tropica*		Southern Iran	JX560467.1
*L. tropica*		Southern Iran	JX560469.1
*L. tropica*		Southern Iran	JX560470.1
*L. tropica*		Southern Iran	JX560482.1
*L. tropica*	ISER/IL/98/LRC-L747	Israel	LC459350.1
*L. tropica*	**SA-Lt44**	**Eastern Saudi Arabia**	OK560763.1
*L. tropica*	**SA-Lt77**	**Eastern Saudi Arabia**	OK560795.1
*L. tropica*	**SA-Lt78**	**Eastern Saudi Arabia**	OK560796.1
*L. tropica*		Malaysia	OL413428.1
*L. tropica*	**MHOM/SA/2021/G02**	**Southern Saudi Arabia**	ON872294.1
*L. tropica*	**MHOM/SA/2021/G71**	**Southern Saudi Arabia**	ON872333.1
*L. tropica*	**MHOM/SA/2021/G58**	**Southern Saudi Arabia**	ON872334.1
*L. tropica*	**MHOM/SA/2021/G51**	**Southern Saudi Arabia**	ON872335.1
*L. tropica*	**MHOM/SA/2021/G82**	**Southern Saudi Arabia**	ON872336.1
*L. tropica*	**MHOM/SA/2021/G08**	**Southern Saudi Arabia**	ON872354.1
*L. tropica*	**MHOM/SA/2021/G54**	**Southern Saudi Arabia**	ON872355.1
*L. tropica*	**MHOM/SA/2021/G16**	**Southern Saudi Arabia**	ON872356.1
*L. tropica*	**MHOM/SA/2021/G67**	**Southern Saudi Arabia**	ON872357.1
*L. tropica*	**MHOM/SA/2021/G32**	**Southern Saudi Arabia**	ON872358.1
*L. tropica*	**MHOM/SA/2021/G47**	**Southern Saudi Arabia**	ON872359.1

**Table 2:** Number of cutaneous leishmaniasis cases based on demographic information in Asir during 2021.

Characteristic	Female, N = 73 ^1^	Male, N = 121 ^1^	*p*-Value ^2^	Characteristic	Female, N = 73 ^1^	Male, N = 121 ^1^	*p*-Value ^2^
Nationality			<0.001	Age_group			<0.001
Non-Saudi	0 (0%)	18 (15%)		0–5	15 (21%)	18 (15%)	
Saudi	73 (100%)	103 (85%)		6–12	18 (25%)	23 (19%)	
Lesion Number			0.041	13–15	5 (6.8%)	4 (3.3%)	
Multiple	24 (33%)	24 (20%)		16–18	1 (1.4%)	4 (3.3%)	
Single	49 (67%)	97 (80%)		19–24	9 (12%)	9 (7.4%)	
Lesion Location			0.8	25–29	4 (5.5%)	16 (13%)	
Abdomen	2 (2.7%)	3 (2.5%)		30–39	6 (8.2%)	14 (12%)	
Face	42 (58%)	77 (64%)		40–49	4 (5.5%)	12 (9.9%)	
Feet-Leg	8 (11%)	11 (9.1%)		50–59	5 (6.8%)	10 (8.3%)	
Hand-Arm	21 (29%)	30 (25%)		60+	6 (8.2%)	11 (9.1%)	
Lesion Appearance			>0.9	Lesion size			0.8
Dry	71 (97%)	118 (98%)		<1 cm	6 (8.2%)	10 (8.3%)	
Wet	2 (2.7%)	3 (2.5%)		1–3 cm	56 (77%)	97 (80%)	
				4–5 cm	11 (15%)	14 (12%)	

^1^ n (%). ^2^ Pearson’s Chi-squared test; Fisher’s exact test.

## Data Availability

The data in this study are available on request from Y.A.
